# LLT1 and CD161 Expression in Human Germinal Centers Promotes B Cell Activation and CXCR4 Downregulation

**DOI:** 10.4049/jimmunol.1502462

**Published:** 2016-02-01

**Authors:** Alba Llibre, Constantino López-Macías, Teresa Marafioti, Hema Mehta, Amy Partridge, Carina Kanzig, Felice Rivellese, Jacob D. Galson, Lucy J. Walker, Paul Milne, Rodney E. Phillips, Dominic F. Kelly, Gordon J. Freeman, Mohey Eldin El Shikh, Paul Klenerman, Christian B. Willberg

**Affiliations:** * Peter Medawar Building for Pathogen Research, University of Oxford, Oxford OX1 3SY, United Kindgom;; † Medical Research Unit on Immunochemistry, Specialties Hospital, National Medical Centre “Siglo XXI,” Mexican Institute for Social Security, 06720 Mexico City, Mexico;; ‡ Department of Histopathology, University College London, London WC1E 6JJ, United Kingdom;; § Centre for Experimental Medicine and Rheumatology, William Harvey Research Institute, Barts and the London School of Medicine and Dentistry, Queen Mary University of London, London EC1M 6BQ, United Kingdom;; ¶ Oxford Vaccine Group, Department of Paediatrics, National Institute for Health Research, Oxford Biomedical Research Centre, University of Oxford, Oxford OX3 7LJ, United Kingdom;; ‖ Newcastle University, Newcastle upon Tyne NE1 7RU, United Kingdom;; # Department of Medical Oncology, Dana-Farber Cancer Institute, Harvard Medical School, Boston, MA 02215; and; ** Oxford National Institute for Health Research Biomedical Research Centre, Oxford OX3 9DU, United Kingdom

## Abstract

Germinal centers (GCs) are microanatomical structures critical for the development of high-affinity Abs and B cell memory. They are organized into two zones, light and dark, with coordinated roles, controlled by local signaling. The innate lectin-like transcript 1 (LLT1) is known to be expressed on B cells, but its functional role in the GC reaction has not been explored. In this study, we report high expression of LLT1 on GC-associated B cells, early plasmablasts, and GC-derived lymphomas. LLT1 expression was readily induced via BCR, CD40, and CpG stimulation on B cells. Unexpectedly, we found high expression of the LLT1 ligand, CD161, on follicular dendritic cells. Triggering of LLT1 supported B cell activation, CD83 upregulation, and CXCR4 downregulation. Overall, these data suggest that LLT1–CD161 interactions play a novel and important role in B cell maturation within the GC in humans.

## Introduction

The germinal center (GC) reaction is critical for long-lasting protection against pathogens. GCs are the anatomical sites within secondary lymphoid organs where B cells proliferate and mutate their BCRs to be selected according to their affinity for Ag. Two distinct areas with different functions can be identified within the GC; these are the dark zone (DZ) and the light zone (LZ). In the former, B cells proliferate and hypermutate their BCRs to generate Ab variation, whereas the quality of these BCRs is assessed in the latter, ultimately leading to selection of high-affinity B cell clones (1, 2). DZ B cells are characterized by being CD83^low^CXCR4^high^, whereas LZ B cells are CD83^high^CXCR4^low^ (3). B cells that have successfully competed for Ag develop into clones and exit the GC expressing high-affinity Abs and long-lived memory. Thus, this process is crucial to vaccinology. At the same time, however, as a site of mutation and proliferation, aberrant reactions can lead to the development of B cell lymphomas and autoimmunity. Understanding the mechanisms that drive this process has significant implications in health care.

C-type lectin-like receptors (CLRs) are encoded in the NK gene complex (NKC) and can be expressed in a wide range of human cell types, including NK cells. They are particularly relevant in the context of innate immune responses. The CLRs lectin-like transcript 1 (LLT1) and CD161 are genetically linked physiological binding partners, located adjacent to one another within the NKC (4–7). Structurally, LLT1 shares the greatest homology with the other C-type lectins activation-induced C-type lectin and CD69 (8). Within murine models, LLT1 shows a similar expression pattern to MHC class I (9, 10), whereas in humans it is limited to activated lymphocytes and monocytes (8, 11–13) and recently on respiratory syncytial virus–infected primary human bronchial epithelial cells (14), although the published literature presents some inconsistencies. In contrast, the expression of LLT1’s binding partner, CD161, has been relatively well characterized, delineating a family of innate-like T lymphocytes and NK cells (15). Functional studies have described inhibitory and activating roles for both molecules (6, 7, 15–23). These studies suggest that interactions between LLT1 and CD161 can result in bidirectional signaling and have functional consequences for both cells involved.

In this study, we show the high expression of LLT1 on human GC B cells and GC-derived B cell lymphomas, extending previous studies (6, 8, 11–13, 17). We also show that LLT1 expression remains on early plasmablasts, but is absent from memory B cells and plasma cells. The LLT1 ligand, CD161, was found, unexpectedly, on follicular dendritic cells (FDCs). Finally, triggering of LLT1 promoted the upregulation of CD83 on B cell and drives DZ B cells toward a LZ phenotype through the downregulation of CXCR4. Previously, LLT1 and CD161 were considered part of innate immune responses. The present study demonstrates a functional role for an innate receptor pairing at the heart of a critical adaptive immune process, the GC reaction in humans.

## Materials and Methods

### Tissues, cells, and cell lines

Human tonsillar tissue was obtained following routine tonsillectomy from the files of the Department of Cellular Pathology (University College London Hospital, London, U.K.); Human Tissue Resource Centre, Barts and the London National Health Service Trust, Queen Mary School of Medicine and Dentistry; and from the Ear, Nose, and Throat Department, John Radcliffe Hospital, Oxford, U.K. Normal tonsillar tissue sections were obtained from ProteoGenix (Schiltigheim, France). Tonsil-derived single cells were collected by mechanical disruption of tonsil samples or collagenase D (1 mg/ml; Boehringer Mannheim) and DNase I (0.1 mg/ml; Sigma-Aldrich, Dorset, U.K.) digestion, as stated. The lymphoma samples analyzed were in the form of 0.6- to 1-mm core tissue arrays.

PBMCs obtained from the National Blood Transfusion Service (National Health Service Blood and Transplant) were isolated on a Lymphoprep gradient (Axis-Shield, Dundee, U.K.).

Bulk B cells were isolated by negative selections from PBMCs or tonsils by magnetic isolation (Stemcell Technologies, Cambridge, U.K.) following manufacturer’s protocols.

300.19-hCLEC2D cells were created by transfection of 300.19 with a vector expressing human CD161/CLEC2D cDNA and maintained under selection.

Vaccine samples were obtained from the Oxford Vaccine Group, Churchill Hospital (Oxford, U.K.) following HBV vaccinations.

Bone marrow samples were obtained from routine hip joint operations (Newcastle University, Newcastle upon Tyne, U.K.). Samples were filtered (40 mm), washed with PBS, homogenized, isolated on a Lymphoprep gradient (Axis-Shield) and aliquoted in FCS plus 10% DMSO (Sigma-Aldrich) and stored in liquid nitrogen until required.

For sorted cells, DZ B cells were FACS sorted using a MoFlo XDP (Beckman Coulter, High Wycombe, U.K.) from purified B cells and defined as singlets, alive, CD38^mid^IgD^−^CXCR4^high^CD83^low^ cells.

Informed consent was obtained from all subjects.

### In vitro differentiation of plasma cells

Memory B cells were differentiated in vitro into plasmablasts and plasma cells, following the protocol described in Jourdan et al. (24). Briefly, CD27^+^ Memory B cells were first purified as bulk B cells by negative selection from PBMCs by magnetic isolation followed by positive selection for CD27^+^ cells (Stemcell Technologies) following the manufacturer’s protocol. The cells were then seeded at 1.5 × 10^5^ cells/ml and cultured for 4 d in the presence of CpG oligodeoxynucleotide 2006 (10 μg/ml; Invitrogen, Paisley, U.K.), soluble human his-rCD40L (50 ng/ml; R&D Systems, Abingdon, U.K.) and anti–poly-his (5 μg/ml; R&D Systems), IL-2 (20 U/ml; R&D Systems), IL-10 (50 ng/ml; Miltenyi Biotec, Surrey, U.K.), and IL-15 (10 ng/ml; Miltenyi Biotec). At day 4 of culture, the cells were washed and cultured at 2.5 × 10^5^ cells/ml with IL-2 (20 U/ml; Miltenyi Biotec), IL-10 (50 ng/ml), IL-15 (10 ng/ml; PeproTech, London, U.K.), and IL-6 (50 ng/ml; Miltenyi Biotec). At day 7 of culture, cells were washed and seeded with IL-6 (50 ng/ml; PeproTech), IL-15 (10 ng/ml; PeproTech), and IFN-α (500 U/ml; R&D Systems) for 3 d.

### Flow cytometry

FACS staining was external unless otherwise specified. Cells were stained with a Live/Dead fixable near-IR dead cell stain kit, for 633 or 635 nm excitation (Invitrogen). For internal staining, cells were fixed with 2% formaldehyde (Sigma-Aldrich) and permeabilized with (×10) permeabilization buffer (eBioscience, Hatfield, U.K.). The following Abs were used for flow cytometric analysis: anti–CD4-PerCp-Cy5.5 (RPA-T4), anti–CD19-Pacific Blue (HIB19), anti–CD38-PerCP-Cy5.5 (HIT2), anti–IgD-FITC (IA6-2), anti–CXCR5-Pacific Blue (J252D4), anti–CD27-PE-Cy7 (M-T271), anti–CD34-FITC (561), anti–CD68-allophycocyanin (Y1/82A), anti–CD83-BV421 (HB15e), anti–ICOS-allophycocyanin (C398.4A) (BioLegend, London, U.K.), anti–CD20-Pacific Orange (HI47, Life Technologies, Paisley, U.K.), anti–CD138-allophycocyanin (B-A38, Beckman Coulter), anti–CD10-PE-Cy7 (HI10a), anti–CD45-RA-FITC (T6D11), anti–CD161-PE (191B8), anti–IgM-allophycocyanin (PJ2-22H3), anti–CXCR4-allophycocyanin (12G5, Miltenyi Biotec), anti–CD3-FITC (ICHT1, R&D Systems), anti–LLT1-PE (402659, R&D Systems), anti–IgG isotype control-PE, IgG1 and IG2A isotype controls (R&D Systems), and anti–mouse IgG-PE (R&D Systems).

Novel mouse anti-human CLEC2D (LLT1) mAbs were generated, including clones 359.7G7 (mIgG1) and 359.2H7 (mIgG2al). All purified Abs are dialyzed against PBS, are low in endotoxin (<2 endotoxin units/mg), and are filtered sterile.

### Immunohistochemistry

Tissue deparaffinization was performed using Histo-Clear (National Diagnostics, Hull, U.K.) and ethanol (Sigma-Aldrich; 100, 90, and 70%). Heat-mediated Ag retrieval was achieved using Dako target retrieval solution (Dako, Ely, U.K.). Endogenous peroxidase activity was blocked using 3% H_2_O_2_ (5 min × 2; Alfa Aesar, Heysham, U.K.) and 0.1% sodium azide (15 min; Sigma-Aldrich) in water. Nonspecific binding was blocked with 0.5% blocking reagent (PerkinElmer, Beaconsfield, U.K.) in PBS.

Cy5/fluorescein stains were amplified using amplification reagents (PerkinElmer) diluted in tyramide amplification buffer (12 ml 500 mM Tris, 18 ml H_2_O, 20 mg imidazole [Sigma-Aldrich], 1 μl H_2_O_2_). Residual peroxidase activity was blocked by incubating the slides in 3% H_2_O_2_ and then 0.1% sodium azide in water. Slides were mounted with ProLong Gold antifade reagent with DAPI (Invitrogen).

A goat polyclonal anti-LLT1 Ab (AF3480, R&D Systems) and a novel mouse mAb (7G7) were used for immunohistological staining, as well as CD3 (clone LN10, Leica Microsystems, Cambridge, U.K.) and PAX-5 (clone 24, BD Biosciences, Oxford, U.K.).

Anti-CD161 (B199.2, AbD Serotec, Kidlington, U.K.; 1:100), IgG1, or IgG2A isotype controls (R&D Systems) were also used and incubated overnight at 4°C and then incubated with anti-goat/mouse polymer (Vector Laboratories, Peterborough, U.K.) HRP conjugated (Vector Laboratories), followed by ImmPACT diaminobenzidine peroxidase substrate (Vector Laboratories). The section was covered with hematoxylin (Vector Laboratories) and washed.

In addition to these stains, the following Abs were used for fluorescence immunohistochemistry: anti-LLT1 goat polyclonal (AF3480, R&D Systems, 1:200), anti-CD68 (1:200, clone PG-M1), anti-FDC (1:100, clone CNA.42), anti-CD19 (1:500, clone LE-CD19), anti-CD20cy (1:1000, clone L26), anti-CD3 (1:200, clone F7.2.38), anti-CD79α (1:200, clone JCB117), anti-CD45 (1:1000, clone 2B11 plus PD7/26), anti-IgG (1:1000, rabbit), anti-IgM (1:1000), anti-CD38 (1:200, clone At13/5), anti-CD138 (1:100, clone MI15), anti-Ki67 (1:200, clone MIB-1), and anti–IFN regulatory factor (IRF) 4 (1:200, clone MUM1) (Dako); anti–cathepsin G (1:1000), anti-IgD (1:200), anti-IgA (1:300) (Leica Biosystems, Newcastle upon Tyne, U.K.); anti-IgE (rabbit, Abcam, Cambridge, U.K.), and normal goat IgG and mouse IgG1/IgG2A isotype controls (R&D Systems). Abs were mouse derived unless otherwise specified.

For the FDC and CD161 costaining in frozen tonsils, four different anti-CD161 Abs were used: 191B8 (Miltenyi Biotec), IMG141F1F1 (Abcam), B199.2 (AbD Serotec), and HP-3G10 (BioLegend). Ten-micrometer-thick frozen tonsillar sections were labeled with 10 μg/ml goat anti-LLT1 (R&D Systems, AF3480-SP), mouse anti-CD161 (Abcam, ab23624), and rabbit anti-CD3 (Dako, A0452), followed by 10 μg/ml donkey anti–goat-Alexa Fluor 488, donkey anti–mouse-Alexa Fluor 594, and donkey anti–rabbit-Alexa Fluor 647 (Jackson ImmunoResearch, Suffolk, U.K.), respectively, and peanut agglutinin (PNA; Vector Laboratories, Peterborough, U.K.).

### B cell activation

Isolated B cells were stimulated with oligodeoxynucleotide 2006 (CpG, InvivoGen, Wiltshire, U.K.) for 48 h. TLR signaling inhibition studies used the MyD88 inhibitory peptide Pepinh-MYD at the given concentration (InvivoGen). B cell stimulations were achieved using goat anti-human IgM/G/A F(ab′)_2_ (Jackson ImmunoResearch) fragments at 1 μg/ml, anti-CD40 (clone S2C6, Macbeth) at 5 μg/ml, or with IL-4 (PeproTech), IL-10, IL-21 (Miltenyi Biotec), PGE_2_ (Sigma-Aldrich), IL-15 (Miltenyi Biotec), and BAFF (Miltenyi Biotec) (all at 50 ng/ml). Proliferations assays were performed using a CellTrace Violet (CTV) cell proliferation kit (Life Technologies).

The CD161 blocking assay included 2 × 10^6^ B cells/ml in 500 μl in a 48-well plate (Corning) with combinations of CpG (5 μM), anti-CD40 (5 μg/ml) plus IL-4 (50 ng/ml), anti-CD161 at 1 μg/ml (clone 191.B8, Miltenyi Biotec), and IgG2A isotype control (R&D Systems).

For LLT1 crosslinking, recombinant CD161 (R&D Systems) or IgG1 isotype control (R&D Systems) were bound to a 96-well ELISA plate (Greiner Bio-One, Stonehouse, U.K.) overnight prior to the addition of B cells and BCR stimulus as described above.

### Proliferation assay

Proliferation assays were performed using a CTV cell proliferation kit (Life Technologies). Purified B cells were stained with 2.5 μM CTV for 10 min at room temperature, following the manufacturer’s instructions. Cells were then counted and 2 × 10^5^ B cells were plated per well in a U-bottom 96-well plate with CpG (0.5 μg/ml) for 5 d.

### CD161 depletion

Tonsillar cells were stained with CD161-PE (Miltenyi Biotec) and depleted from the sample by FACS sorting using a MoFlo (Beckman Coulter). After CpG (0.5 μM, InvivoGen) or anti-CD40 (5μg/ml, Macbeth) plus IL-4 stimulation (50 ng/ml, PeproTech), levels of CD38 and CD83, respectively, were measured in alive CD19^+^ cells.

### Real-time PCR

For real time RT-PCR, amplification was assessed using molecular probes 32 (for CD161) and 60 (for GAPDH) from the Universal ProbeLibrary set, human (Roche, Welwyn Garden City, U.K.) on a LightCycler 480 (Roche). The following primers were used: CD161, forward, 5′-AAATGCAGTGTGGACATTCAA-3′, reverse, 5′-CTCGGAGTTGCTGCCAATA-3′ (Eurofins MWG Operon, Ebersberg, Germany); GADPH, forward, 5′-AGCCACATCGCTCAGACAC-3′, reverse, 5′-GCCCAATACGACCAAATCC-3′; CXCR4, forward, 5′- GGTGGTCTATGTTGGCGTCT-3′, reverse, 5′-ACTGACGTTGGCAAAGATGA-3′ (Eurofins MWG Operon, Germany).

### Data acquisition and analysis

FACS analysis was performed on Miltenyi Biotec MACSQuant cytometer and analyzed with FlowJo version 9.6.2 software (Tree Star, Ashland, OR). Graphs and statistical analysis were performed using GraphPad Prism version 6.0a (GraphPad Software, San Diego, CA).

For analysis of immunohistochemical staining, images were acquired on a DSS1 Coolscope slide scanner (Nikon, Kingston upon Thames, U.K.).

For immunofluorescent microscopy, images were acquired on an Olympus Fluoview FV1000 microscope (Olympus, Southend-on-Sea, U.K.) and analyzed using Fiji (ImageJ, National Institutes of Health, Bethesda, MD). The Leica TCS-SP2 AOBS confocal laser-scanning microscope was used for the CD161 immunofluorescence, and the images were captured and processed with Leica confocal software (LCS Lite) and ImageJ.

## Results

### LLT1 is highly expressed on GC B cells

**FIGURE 1.**
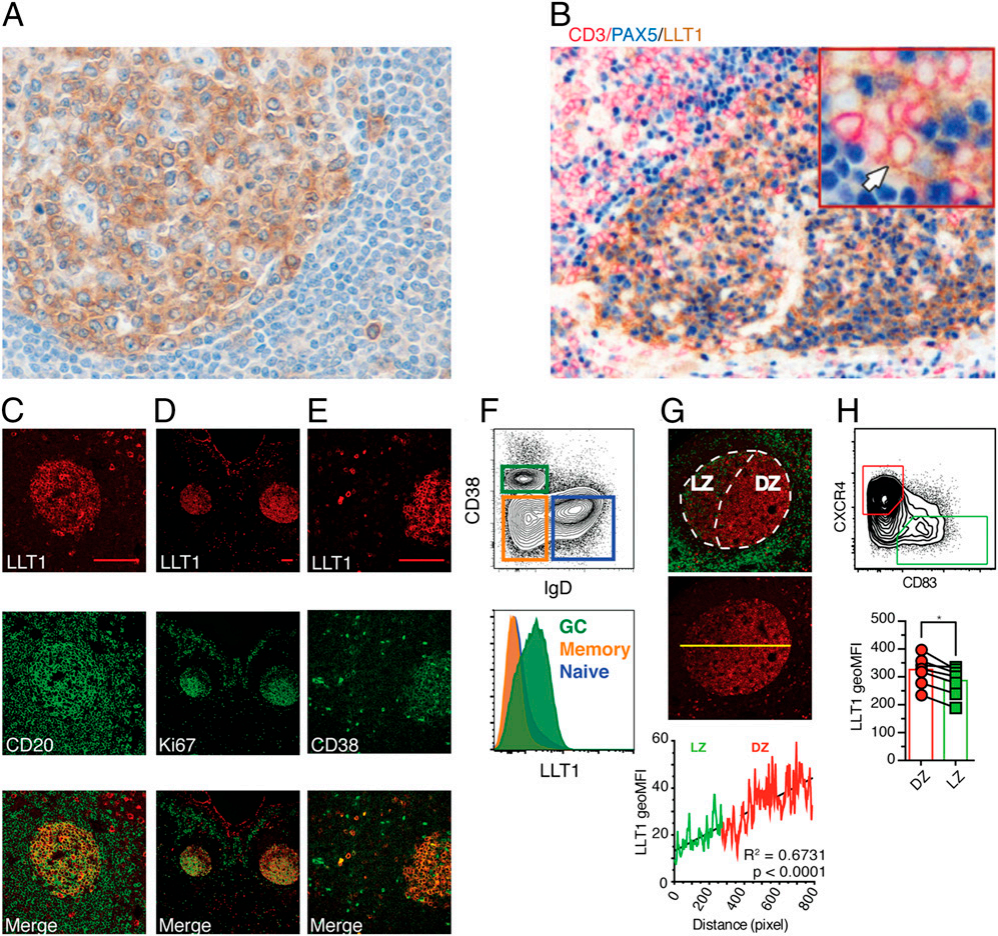
LLT1 is expressed on GC B cells. (**A**) Expression of LLT1 (brown), using the 7G7, was assessed in formalin-fixed, paraffin-embedded human tonsillar tissue sections (original magnification ×40) (representative of five sections). (**B**) Triple staining for LLT1 (membrane and cytosol brown), CD3 (membrane pink), and PAX-5 (nuclear blue) showed LLT1 (goat-polyclonal, brown) strongly associated with PAX-5 and only rarely with CD3 (arrow in *inset*) (original magnification ×20, *inset* ×60). Costaining of LLT1 (goat-polyclonal) with CD20 (**C**), Ki67 (**D**), and CD38 (**E**) (scale bar, 100 μm, representative of three sections). (**F**) FACS analysis of LLT1 expression on naive (blue), memory (orange), and GC (green) B cells (representative plot of five independent experiments). (**G**) Cross-sectional analysis through a region (yellow band) of a GC (green indicates CD3, red indicates LLT1; representative of three independent experiments). Geometric mean fluorescence intensity (geoMFI) of LLT1 expression within the defined yellow region plotted against pixel count; the LZ (green) and DZ (red) regions are highlighted. (**H**) FACS analysis of LLT1 (2H7 clone) on LZ and DZ, CXCR4 and CD83, respectively (*p < 0.05, nonparametric paired *t* test).

LLT1 expression was observed within normal human tonsils and predominantly expressed within GCs (Fig. 1A). This was demonstrated by immunohistological staining using two independent Abs: a commercial goat polyclonal anti-LLT1 Ab, and a novel anti-LLT1 Ab (clone 7G7, see *Materials and Methods*, Supplemental Fig 1A, 1B). Furthermore, this was not restricted to tonsillar tissue, as GCs within spleen, lymph nodes, and Peyer’s patches were also LLT1^+^ (Supplemental Fig. 1C–F).

LLT1 expression within GC cells was restricted to PAX-5^+^ cells, B cells (Fig. 1B). Intra-GC T cells (Fig. 1B), tingible body macrophages, neutrophils, and FDCs within secondary follicles were all negative for LLT1 (data not shown). However, within the interfollicular areas, LLT1 expression was seen on a small proportion of CD3^+^ T cells (Fig. 1B, *inset*). Furthermore, LLT1 expression on GC B cells was confirmed by costaining for CD20 (Fig. 1C), Ki67 (Fig. 1D), and CD38 (Fig. 1E). FACS analysis, using a second novel anti-LLT1 Ab (clone 2H7), also confirmed LLT1 on GC B cells, defined as CD38^mid^IgD^−^, as previously described (25–27) (Fig. 1F).

LLT1 expression was not homogeneous throughout the GC. A clear difference in LLT1 staining intensity could be seen by immunohistology. Cross-sectional analysis through the GC revealed increased LLT1 levels within the DZ compared with the LZ (defined by the presence of CD3^+^ cells, stained green, Fig. 1G). Moreover, FACS analysis confirmed higher LLT1 expression on DZ B cells (CD83^low^CXCR4^high^) compared with LZ B cells (CD83^high^CXCR4^low^) (Fig. 1H).

### LLT1 is expressed on plasmablasts but not memory B cells

**FIGURE 2.**
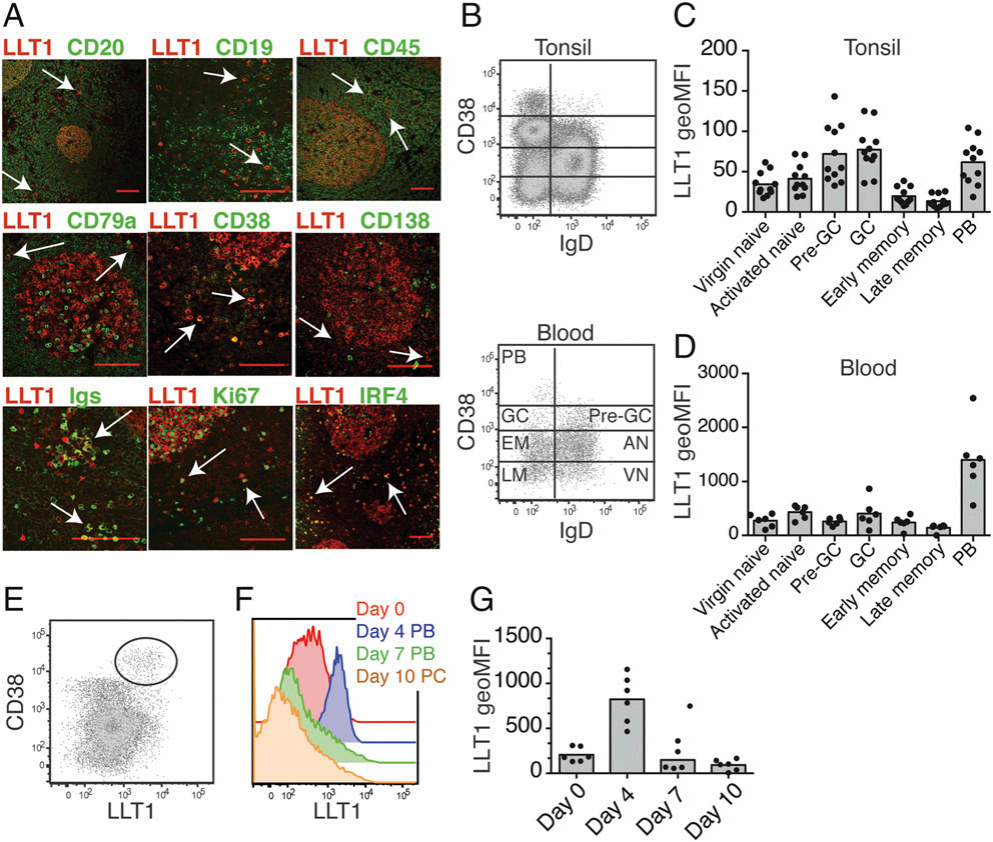
LLT1 is expressed on early plasmablasts. (**A**) Costainings in formalin-fixed, paraffin-embedded human tonsillar tissue of LLT1 with CD20 (original magnification ×10), CD19 (original magnification ×20), CD45 (original magnification ×10), CD79a (original magnification ×20), CD38 (original magnification ×20), CD138 (original magnification ×20), Igs (original magnification ×20), Ki67 (original magnification ×20), and IRF4 (original magnification ×10). White arrows indicate double-positive cells (scale bars, 100 μm), representative of three independent sections. (**B**) Seven different B cell subsets identified by IgD and CD38 expression in tonsillar tissue (*top*) and PMBCs (*bottom*): virgin naive (VN), activated naive (AN), pre-GC, early memory (EM), late memory (LM), and plasmablasts (PB) are shown. Geometric mean fluorescence intensity (geoMFI) of LLT1 was measured in the subsets: tonsils (**C**, *n* = 10) and blood (**D**, *n* = 3). (**E**) Expression of LLT1 in the plasmablast subset in an example vaccinated individual 1 wk postimmunization, representative of eight similar individuals. (**F** and **G**) LLT1 expression in in vitro–generated plasmablasts analyzed on day 0, day 4 plasmablasts (PB), day 7 PB, and day 10 plasma cells (PC). (F) Representative FACS plot of time course. (G) Graphed data pooled from three independent experiments (total *n* = 6).

B cells exit the GC upon differentiation into plasma or memory B cells. Whether LLT1 expression is maintained in these GC B cell derivatives is not known; therefore, we sought to track LLT1 expression through these stages. Plasmablasts were identified by costaining tonsillar sections with LLT1 plus CD19, CD20, CD38, CD45, CD79α, CD138, IRF4 (MUM-1), and Igs (Fig. 2A). LLT1^+^ cells coexpressed these markers in a pattern suggestive of a plasmablast phenotype (CD45^low^, CD20^−^, CD19^low^, CD79α^−/low^, pan Ig^+^, MUM-1^+/−^, CD38^+^, Ki67^+/−^, CD138^−^), although there remained a number of LLT1^+^ cells that did not fit this phenotype and remain to be identified.

FACS analysis, using the Bm1–Bm5 classification based on CD38 and IgD markers (Fig. 2B), revealed that LLT1 expression was restricted to GC B cells, pre-GC B cells, and plasmablasts within tonsillar-derived cells (Fig. 2C) and plasmablasts within PBMCs (Fig. 2D) (25, 26, 28). The IgD^+^CD38^low^ compartment, comprising both naive and transitional B cells, were mainly negative for LLT1 (Fig. 2C, 2D) (29). As plasmablast frequencies are normally low in circulation, we also confirmed LLT1 expression on plasmablasts in individuals 1 wk postvaccination, where plasmablasts are readily detectable (30, 31) (Fig. 2E). LLT1 expression, however, was not detected on plasma cells or memory B cells (Fig. 2A–C).

To confirm that LLT1 is downregulated as plasmablasts mature into plasma cells, we differentiated CD27^+^ memory B cells into plasmablasts and then plasma cells in vitro, as described by Jourdan et al. (24). LLT1 expression was measured by FACS analysis at days 0 (memory B cells), 4 (early plasmablasts), 7 (late plasmabalsts), and 10 (plasma cells) (Fig. 2F). Day 0 resting CD27^+^ memory B cells expressed very low levels of LLT1, but LLT1 was highly upregulated at day 4 on early plasmablasts, but downregulated at further stages of differentiation (Fig. 2G). Additionally, we confirmed the absence of LLT1 on plasma cells in vivo by FACS and immunohistological staining of long-lived, bone marrow–resident CD138^+^ cells from bone marrow samples (data not shown).

Given access to bone marrow samples, we took the opportunity to assess expression levels of LLT1 on the early developmental stages of B cells; thus, we were able to assess LLT1 expression throughout the whole life of a B cell (Supplemental Fig. 2).

### Regulation of LLT1 expression on memory B cells

**FIGURE 3.**
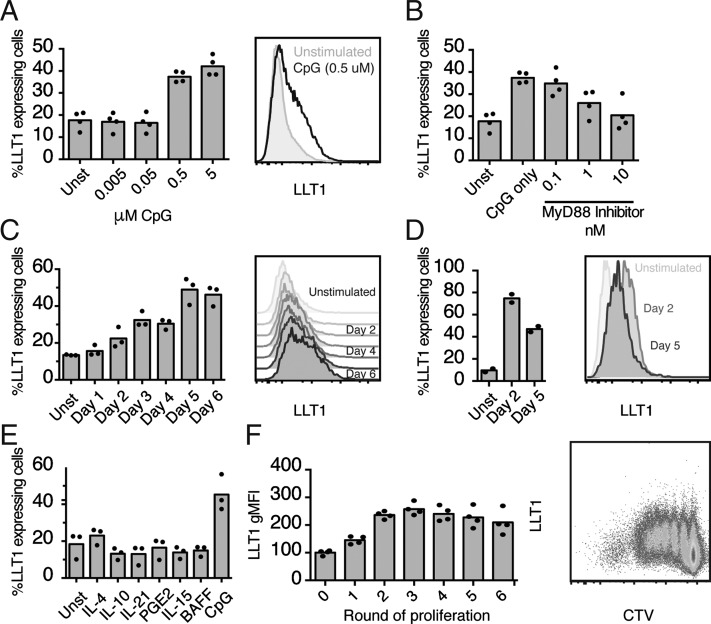
LLT1 is upregulated with CpG, anti-BCR, and anti-CD40 stimulation. (**A**) Purified B cells from PBMCs were stimulated with increasing concentrations of TLR9 agonist CpG for 48 h, which resulted in a dose-dependent increase in the percentage of LLT1-expressing B cells (2H7 clone). (**B**) The effect was blockable with MyD88 inhibitor, Pepinh-MYD, and it was also dose-dependent. (**C**) BCR stimulation resulted in upregulation of LLT1 on B cells. (**D**) LLT1 was also upregulated on B cells upon CD40 stimulation. (**E**) Stimulation with different cytokines (50 ng/ml for 48 h) did not alter expression levels of LLT1 on B cells. (**F**) B cell proliferation, measured by CTV dilution, after 5 d with CpG stimulation (0.5 μM). LLT1 increased in successive rounds of proliferation and reached a plateau. Representative plots of three independent experiments are shown.

Next we questioned how LLT1 was regulated within B cells. Previous work has shown LLT1 can be upregulated following TLR9, BCR, and CD40 stimulation (21). We confirmed CpG-induced expression (Fig. 3A) and extended this to show that this mechanism is MyD88-dependent, as the presence of the MyD88 inhibitor, Pepinh-MYD, blocked upregulation (Fig. 3B). In addition to CpG, we explored the kinetics of both BCR and CD40 stimulation to induced LLT1 expression. BCR signaling in isolated PBMC-derived memory B cells induced a maximum expression after 5 d (Fig. 3C), whereas CD40 stimulation induced LLT1 expression within 2 d (Fig. 3D). Furthermore, B cell activation is associated with a broad range of cytokines; therefore, we assessed the ability of IL-4, IL-10, IL-21, PGE_2_, IL-15, and BAFF to upregulate LLT1. None of the cytokines tested was able to induce LLT1 expression (Fig. 3E) (32–35).

Interestingly, LLT1 expression was associated with early rounds of proliferation after CpG stimulation, increasing with each division until the third, were it plateaued (Fig. 3F). This is consistent with the observation of high LLT1 expression within DZ B cells (Fig. 1G, 1H), plasmablasts (Fig. 2), and B cell lymphomas (see Fig. 6).

### CD161 is expressed on FDCs and on a subset of tonsillar T cells

**FIGURE 4.**
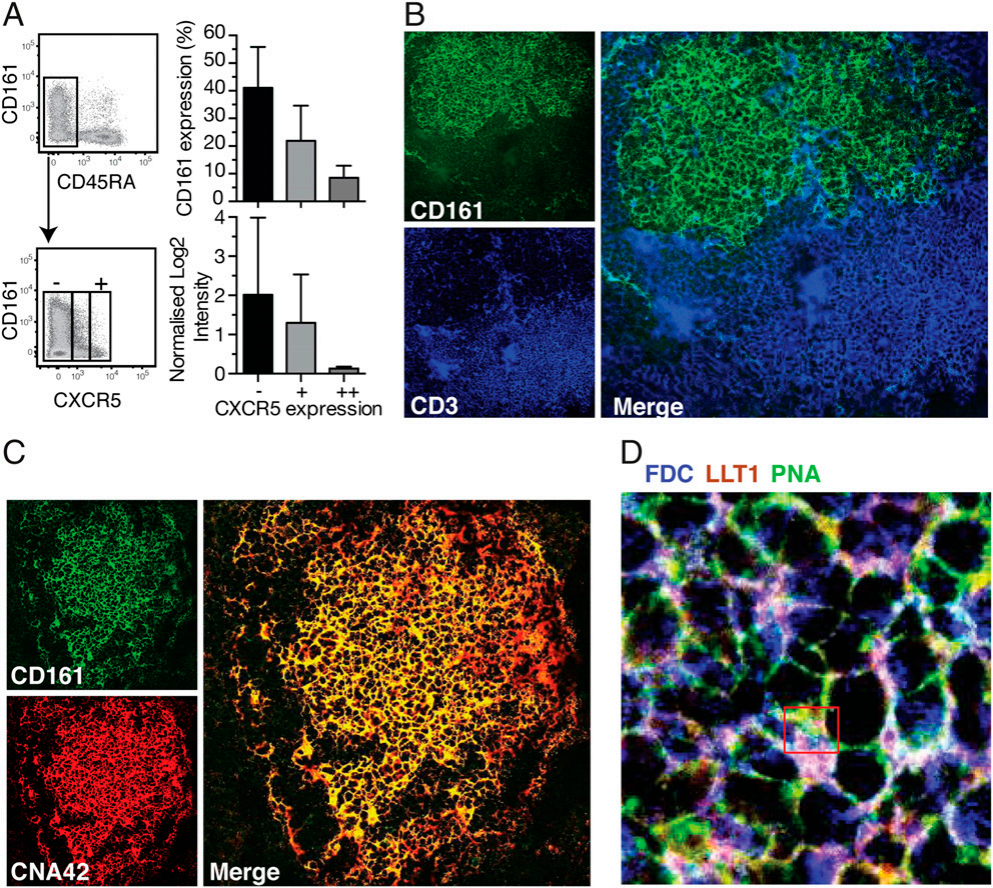
CD161 is expressed on FDCs and on a subset of tonsillar T cells. (**A**) CD161 expression negatively correlated with CXCR5 expression on CD45RA^−^ T cells (CD3^+^CD4+) assessed by FACS (*n* = 5, three independent experiments) and real-time PCR (*n* = 3, two independent experiments). (**B**) Costaining of CD161 and CD3, showing no double-positives within the GC. Original magnification ×20. (**C**) Immunofluorescence staining of frozen human tonsils with an FDC marker (clone CNA.42) and CD161 (clone IMG14F1F11) showed complete overlap between these two markers. Original magnification ×20. (**D**) Triple FDC, LLT1, and PNA stain of a frozen tonsillar human section. Interaction between FDCs and double-positive LLT1-PNA GC B cells can be observed (red square). Representative image of six different follicles. Original magnification ×63 oil objective with 3× digital zoom.

Before we addressed the function of LLT1, we looked for the expression of the LLT1 ligand, CD161, within the context of the GC. CD161 expression has been described on both NK cells and T cells. Therefore, T follicular helper (Tfh) cells were an obvious candidate cell type, with a potential to interact with GC B cells. Flow cytometric analysis of tonsillar T cells revealed a loss of CD161 expression as the Tfh cells progressed to full GC phenotype, as defined by CXCR5 expression (36) (Fig. 4A, *top right panel*). This was confirmed at the mRNA level in sorted tonsillar T cells (Fig. 4A, *bottom right panel*). Although a small fraction (<10%) of CXCR5^high^ Tfh cells expressed CD161, they were not observed within any of the GCs assessed by immunofluorescence microscopy (Fig. 4B, Supplemental Fig. 3). Similarly, within the PBMCs, circulating Tfh cells (defined simply as CXCR5^mid^) (37) also had lower frequencies of CD161^+^ cells compared with the CXCR5^−^ fraction (Supplemental Fig. 3).

Next, CD161 expression was assessed on tonsillar macrophages (including tingible body macrophages), defined by CD68 expression; however, no expression was observed (Supplemental Fig. 3).

Unexpectedly, immunofluorescence staining of human tonsils revealed the expression of CD161 on FDCs (Fig. 4C). Costaining of CD161 and a FDC marker (CNA.42 clone) showed a strong overlap, with a typical stain pattern associated with FDCs. This striking finding was confirmed by histological staining and was not specific to just one clone of anti-CD161 Ab (Supplemental Fig. 3). Costaining of PNA, LLT1, and CD161 showed interaction between LLT1 on GC B cells and CD161, expressed in a FDC pattern, within the GC (Fig. 4D).

### LLT1 promotes B cell activation and downregulation of CXCR4 in GC B cells

Interactions between FDCs and B cells are complex, involving a wide range of receptors (38). Binding of BCRs to Ags, presented by the FDC, and of CD40 to CD40L delivers crucial signals required for B cells to progress to the next stage of the GC reaction and interact with Tfh cells, and subsequently go on to re-enter the DZ (10, 39–41).

**FIGURE 5.**
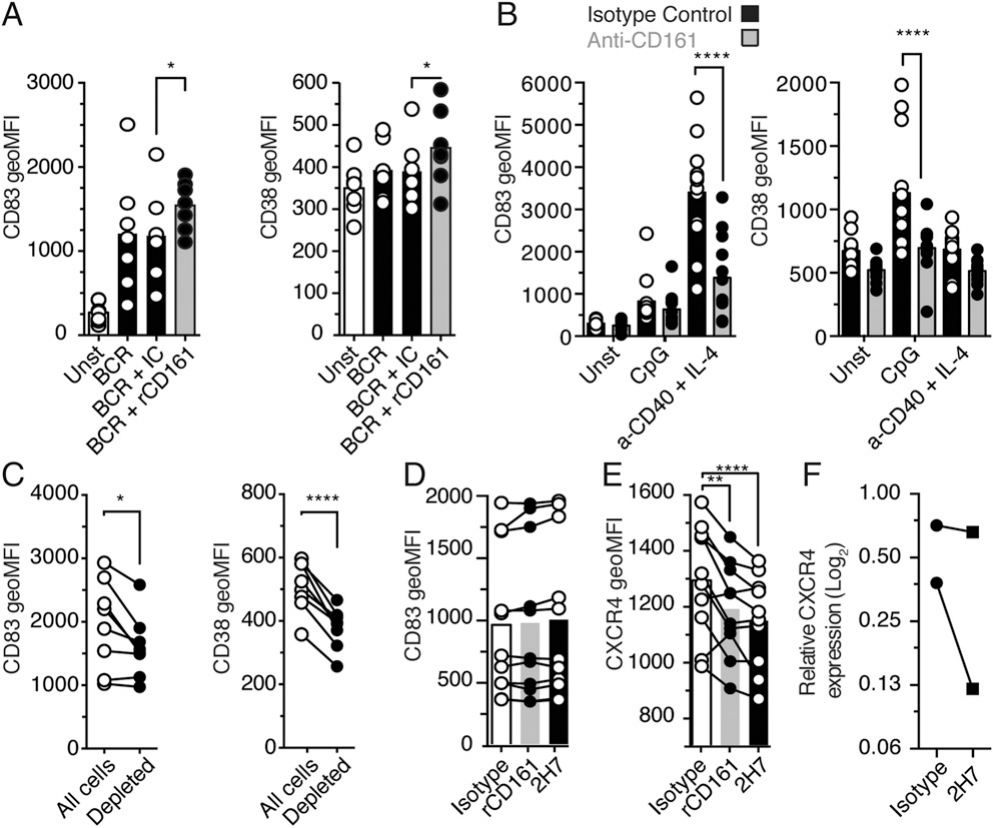
LLT1 promotes B cell activation. (**A**) Purified B cells from PBMCs were stimulated with anti-BCRs only or with anti-BCRs with and without rCD161 or IC. Geometric mean fluorescence intensities (GeoMFI) of CD83 and CD38 were measured by flow cytometry. B cell stimulation with rCD161 resulted in enhanced levels of CD83 and CD38; data were pooled from three independent experiments (*n* = 7, **p < 0.01). (**B**) Tonsillar cells were cultured for 3 d with CpG or anti-CD40 plus IL-4. Blocking LLT1–CD161 interaction resulted in a decreased expression of CD83 and CD38 on B cells; data were pooled from four independent experiments (*n* = 12, ****p < 0.0001). (**C**) Depletion of CD161^+^ cells from tonsillar cells resulted in decreased expression of CD83 (anti-CD40 plus IL-4 stimulation) and CD38 (CpG stimulation) on B cells after 3 d; data were pooled from two independent experiments (*n* = 8; *p < 0.05, ****p < 0.0001, paired *t* test). (**D**) Overnight stimulation of tonsillar B cells with rCD161 or 2H7 anti-LLT1 Ab did not change the expression of CD83 on GC B cells (*n* = 5). (**E**) However, a decrease in CXCR4 expression levels was observed GC B cells (*n* = 10; **p < 0.01, ****p < 0.0001). (**F**) Repeated experiments on sorted DZ B cells revealed a downregulation of CXCR4 mRNA. All statistics were two-way ANOVA using a Bonferroni multiple comparisons test, unless stated otherwise.

As B cell–FDC interactions involve many different ligands and receptors, we explored the role of LLT1 in isolated B cells. Initially, we investigated LLT1 signaling in primary B cells, inducing LLT1 expression through stimulation with anti-BCR, as described above, with and without rCD161 protein or a control protein (IC). After 5 d of stimulation, B cell expression of CD83 and CD38 was measured by flow cytometry. Triggering of LLT1 on B cells through rCD161 resulted in increased B cell activation. Significantly higher increases in CD83 and CD38 were seen on B cells in the presence of rCD161 compared with the controls (Fig. 5A).

As both proteins are highly glycosylated and have a weak binding strength (*K*_d_ of 48 μM), we addressed whether binding of naturally expressed LLT1–CD161 would reveal stronger signaling (42). To analyze this, we cultured tonsillar cells, which included both B cells and CD161^+^ cells, for 3 d with either anti-CD40 plus IL-4 or CpG and assessed their activation state by flow cytometry on day 3. The effect of LLT1 signaling was measured through the use of blocking Abs to CD161 (Fig. 5B, *left panel*). Stimulation of B cells with anti-CD40 plus IL-4 resulted in a striking upregulation of CD83 on day 3, which was significantly inhibited by the addition of the anti-CD161 Ab. This was not observed with the CpG-stimulated sample. In contrast, levels of CD38 expression were highly increased upon CpG stimulation. Once again, the addition of the anti-CD161 blocking Ab significantly reduced CD38 upregulation (Fig. 5B, *right panel*). Furthermore, depletion of CD161^+^ cells from the cellular cultures, prior to stimulation with either anti-CD40 plus IL-4 or CpG, also resulted in a significant decrease in CD83 and CD38 expression, respectively (Fig. 5C, *left panel* for anti-CD40 plus IL-4 stimulation and *right panel* for CpG stimulation).

Finally, we explored the functional consequences of LLT1 triggering on GC B cells. The presence of rCD161 or the crosslinking anti-LLT1 Ab (2H7) did not have an impact on CD83 expression levels (Fig. 5D), CD38, or survival (Supplemental Fig. 4); however, LLT1 signaling resulted in a significant reduction of CXCR4 (Fig. 5E). This was also analyzed on sorted DZ B cells, where the effect of LLT1 crosslinking resulted in the downregulation of CXCR4 at mRNA level (Fig. 5F).

These findings suggest that LLT1 is involved in the transition of DZ B cells to the LZ through the downregulation of CXCR4, while contributing to enhanced levels of CD83 expression.

### LLT1 is expressed in human B cell lymphomas

**FIGURE 6.**
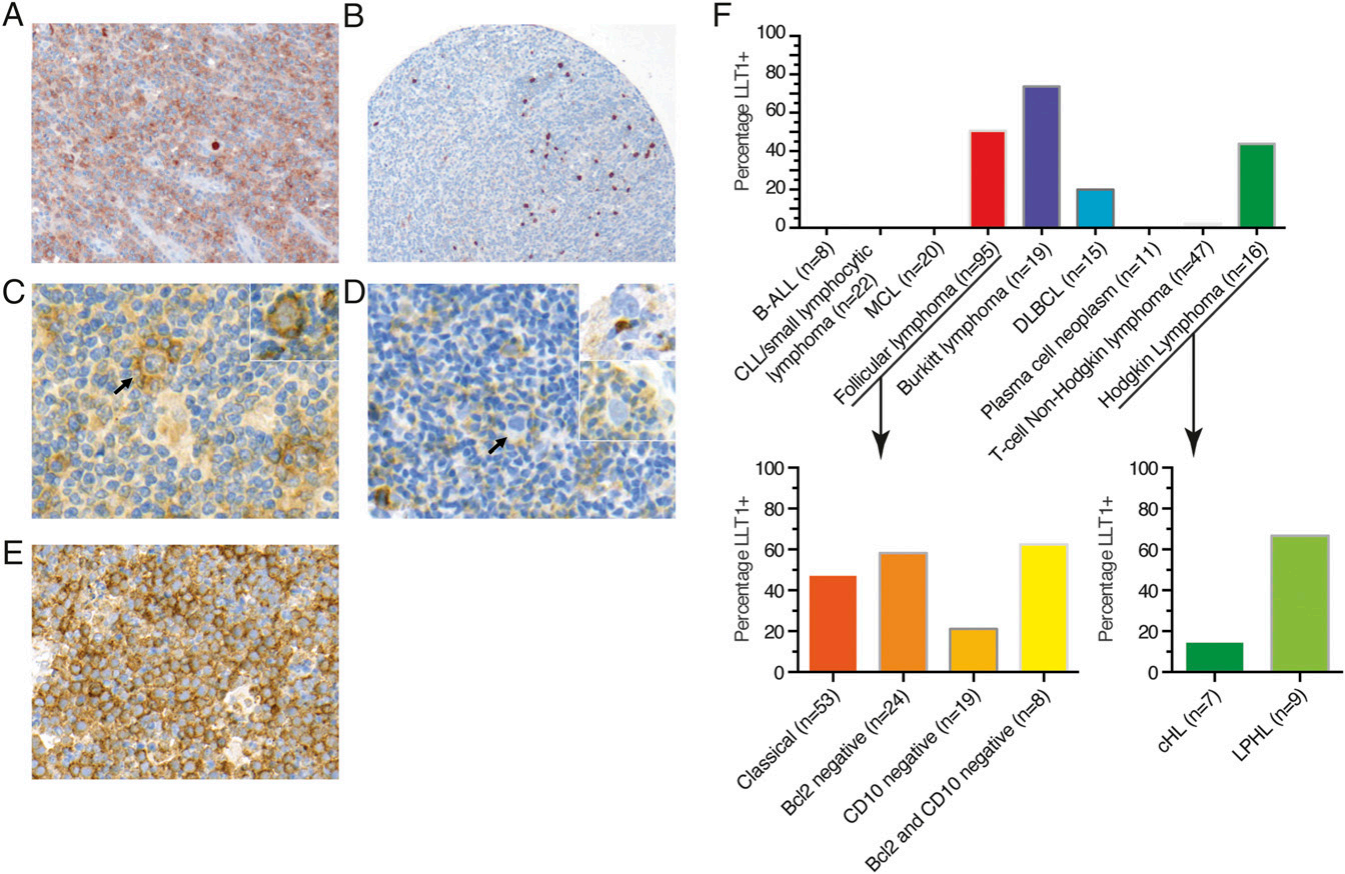
LLT1 is expressed in human B cell lymphomas. (**A**–**E**) Representative stainings of LLT1 (membrane and cytosol brown) as assessed (polyclonal Ab AF3480) in formalin-fixed, paraffin-embedded tissue sections of follicular lymphoma samples [(A) original magnification ×40; (B) original magnification ×10], lymphocyte-predominant Hodgkin lymphoma [(C) original magnification ×40; arrow and *inset* highlighting LLT1^+^ staining, original magnification ×60], classical Hodgkin lymphoma [(D) original magnification ×40; arrow and *inset* highlighting LLT1^+/−^ staining, original magnification ×60], and Burkitt lymphoma [(E) original magnification ×40]. (**F**) The percentage of LLT1^+^ lymphomas was assessed by immunohistochemistry in different B cell non-Hodgkin lymphomas, T cell non-Hodgkin lymphomas, and Hodgkin lymphomas. cHL, classical Hodgkin lymphoma; LPHL, lymphocyte-predominant Hodgkin lymphoma.

As a site of mutation and proliferation, many B cell lymphomas are derived from the GC. Therefore, having established LLT1 expression on GC B cells in vivo under normal conditions, we next examined whether such linked expression was maintained in the context of neoplasia. We assessed LLT1 expression in five B cell lymphoma cell lines. FL18, KHM2, L421, and SUDHL6 were positive for LLT1, whereas Burkitt’s lymphoma–derived Namalwa cells where negative (data not shown). Using a commercially available LLT1 Ab (goat polyclonal, see *Materials and Methods*), LLT1 expression was evaluated in a series of lymphomas (Fig. 6A–E). LLT1 was absent on non-GC–derived lymphomas, such as B-ALL and plasma cell neoplasms. In contrast, LLT1 expression was seen on most Burkitt lymphomas (14 of 19, 73.68%), follicular lymphomas (48 of 95, 51%), and also on Hodgkin lymphomas (7 of 16, 44%). Subdivision of the follicular lymphomas revealed the highest association of LLT1 on atypical phenotypes that have been previously described: Bcl-2^−^ (59%) and Bcl-2^−^ and CD10^−^ (62%) (Fig. 6F, *lower left panel*) (43). Interestingly, LLT1 expression was seen on lymphocyte-predominant Hodgkin lymphomas and not classical Hodgkin lymphomas (Fig. 6F, *lower right panel*). Our data suggest that LLT1 is maintained during malignant transformation in some GC-derived lymphomas and may be a useful additional diagnostic marker.

## Discussion

GC were first characterized >125 years ago. In recent years, our understanding of how these dynamic and complex reactions are orchestrated has advanced rapidly. In this study, we report the expression and function of the innate receptor pairing, LLT1 and CD161, within the GC reaction in humans.

A GC can be divided into two microanatomically distinct regions, the LZ and the DZ. Competition for Ag and Tfh cell help occurs within the LZ, whereas somatic hypermutation and proliferation are restricted to the DZ (reviewed in Ref. 40).

Although LLT1 expression was marginally higher on DZ cells, it was observed on all GC B cells in all lymphoid tissues tested. LLT1 expression was not, however, restricted just to GC B cells, as LLT1^+^ cells were detected outside of the GC. Many of these cells expressed markers associated with a plasmablast phenotype (CD45^low^, CD20^−^, CD19^low^, CD79α^low^, Ig^+^, IRF^+^, CD38^+^, Ki67^+^, CD138^−^), although this does not exclude other cell types. In vitro differentiation of memory B cells into plasmablasts and then plasma cells demonstrated that LLT1 expression was lost as cultures progressed toward terminally differentiated plasma cells at day 10 (24). This was also confirmed on staining bone marrow–derived plasma cells. It appears that LLT1 expression on plasmablasts in vivo is a marker of recent exit from the GC, although whether it can also be independently upregulated remains to be addressed.

It was noted that LLT1 was frequently associated with dividing cell populations both in vivo and in vitro. Zhou et al. (23) also observed that OCIL (LLT1) mRNA expression was at its highest during the osteoblast proliferative phase of bone marrow cultures. In contrast, we observed that LLT1 was absent from plasma cells and both early and late memory B cells, which are nondividing cells. This suggests a link between proliferation and LLT1 expression, although we have not as yet been able to find any functional involvement of LLT1 during proliferation (data not shown). However, LLT1 might be a useful marker for the identification of GC B cells with high-affinity BCRs, as B cells with high-affinity BCRs are able to more efficiently present Ag to Tfh cells and consequently undergo more rounds of division (44).

To better understand the function of LLT1 within the context of the GC, we investigated potential sites of interactions by seeking the expression of its ligand, CD161. Lanier et al. (45) originally identified the expression of CD161 on both CD4^+^ and CD8^+^ T cells, which has been broadened to include innate lymphocyte subsets (15, 46–49). However, murine homologs of CD161, NKR-P1B and NKR-P1D, are not fully analogous, as they differ both in expression profiles and function (reviewed in Ref. 50).

Unexpectedly, CD161 was observed on FDCs. This was particularly surprising, as it is uncommon for NK cell receptors to be expressed on nonhematopoietic cells, although relevant examples exist, for example, KACL on keratinocytes (51) and LLT1 on osteoblasts (23).

Given the close association of CD161 expression and T cells, CD161 expression was also investigated on Tfh cells (CXCR5^mid/high^). However, CD161 expression negatively correlated with CXCR5 expression, at both the protein and mRNA levels. A potential explanation for this may lie in the transcription factor Bcl-6. This master transcription factor drives maturation of T and B cells toward a full GC phenotype. As Bcl-6 expression increases it suppresses other lineage-associated transcription factors, such as retinoic acid–related orphan receptor γT, a transcription factor associated with CD161 expression (46, 52–54). Thus, it is plausible that Bcl-6 inhibits the expression of CD161 through the suppression of retinoic acid–related orphan receptor γT.

The LZ and DZ can be identified by the higher expression of either CD83 or CXCR4, respectively (1, 3, 55–57). Movement between the zones is fundamental for efficient participation within the GC reaction (3, 44, 58). Entry into the DZ requires Tfh interactions and the expression of CXCR4, although CXCR4^−/−^ B cells are still able to transit into a centroblast phenotype and acquire mutations, albeit at a lower level compared with their wild-type counterparts (3, 44, 58). Migration back to the LZ is associated with lower CXCR4 expression, but the underlying mechanisms have not been identified. In this study, crosslinking of LLT1 alone resulted in a reduction in the expression of CXCR4 in DZ B cells. Therefore, these data suggest that LLT1 triggering occurs within the LZ.

Once within the LZ, B cells interact with FDCs and Tfh cells and receive a number of costimulatory signals (59–62). A key receptor in these interactions expressed by the B cells is CD83 (63–65). Triggering of LLT1 alone did not alter the expression of CD83 GC B cells. However, in the context of BCR or CD40 signaling, LLT1 triggering promoted CD83 expression. This suggests that LLT1 may also help enhance B cell interactions with both FDCs and Tfh cells. Combined with the downregulation of CXCR4, these data suggest that LLT1 triggering helps drive B cells through the different GC transitional states within the LZ (57).

LLT1’s high expression within the GC, and its association with proliferating cells, suggests its use as a potential aid for the clinical diagnosis of B cell lymphomas, in particular non–Hodgkin lymphomas, which represent 4–5% of all cancers diagnosed (66). Treatment selection relies on accurate diagnosis, staging of the disease, and identification of adverse prognostic factors. Expressions of Bcl-6, CD10, and IRF4 are used in immunohistochemical stains to separate diffuse large B cell lymphomas into GC and non-GC subtypes (67, 68). However, although immunohistochemistry is widely used, it is not always reliable, in particular when identifying follicular lymphomas with atypical phenotypes. LLT1, therefore, provides a potential new marker for the clinical investigation of lymphomas, and it warrants larger prognostic studies to provide more data on the independent significance of positive or negative stains.

LLT1 and CD161 are both CLRs encoded within the NKC, located adjacent to one another (4–7, 51, 69). Expression of other NKC proteins within the GC is limited to CD69 on both B cell and Tfh cells; however, a function for CD69 within the GC has not been described. The role of particular lectins in the modulation of BCR responses has been explored before. For instance, CD22 and CD72 have been shown to downmodulate BCR-mediated signals (70). Furthermore, the C-type lectin CD23, the low-affinity receptor for IgE, is also relevant in the context of B cell responses (71), as it acts as a ligand for CD21 within the BCR complex. Interestingly, CD23 has been found highly expressed in a proportion of FDCs. Thus, the data presented in this study are consistent with a contribution of C-type lectins to B cell responses through FDC–B cell interactions. This is particularly meaningful, as CLRs, once thought to be essentially involved in innate immunity, have now been shown to play a prominent role in the GC reaction, a hallmark of adaptive immunity.

The data presented in the present study suggest a role for innate components of the human immune system, such as LLT1 and CD161, in the GC reaction. Our data suggest that LLT1 triggering may play a key role in the GC reaction promoting B cell activation and downregulating CXCR4, thus helping to promote an LZ phenotype and facilitating both FDC and Tfh interactions.

## Supplementary Material

Data Supplement
