# Supplementary material for: LLT1 and CD161 Expression in Human Germinal Centers Promotes B Cell Activation and CXCR4 Downregulation
Source: J Immunol. 2016 Feb 1;196(5):2085–94. doi: 10.4049/jimmunol.1502462 (PMC4760235; doi:10.4049/jimmunol.1502462)
Supplement: Data Supplement [file JI_1502462.zip › JI_1502462_Supplemental_Figures_1.pdf]

SUPPLEMENTAL FIGURES

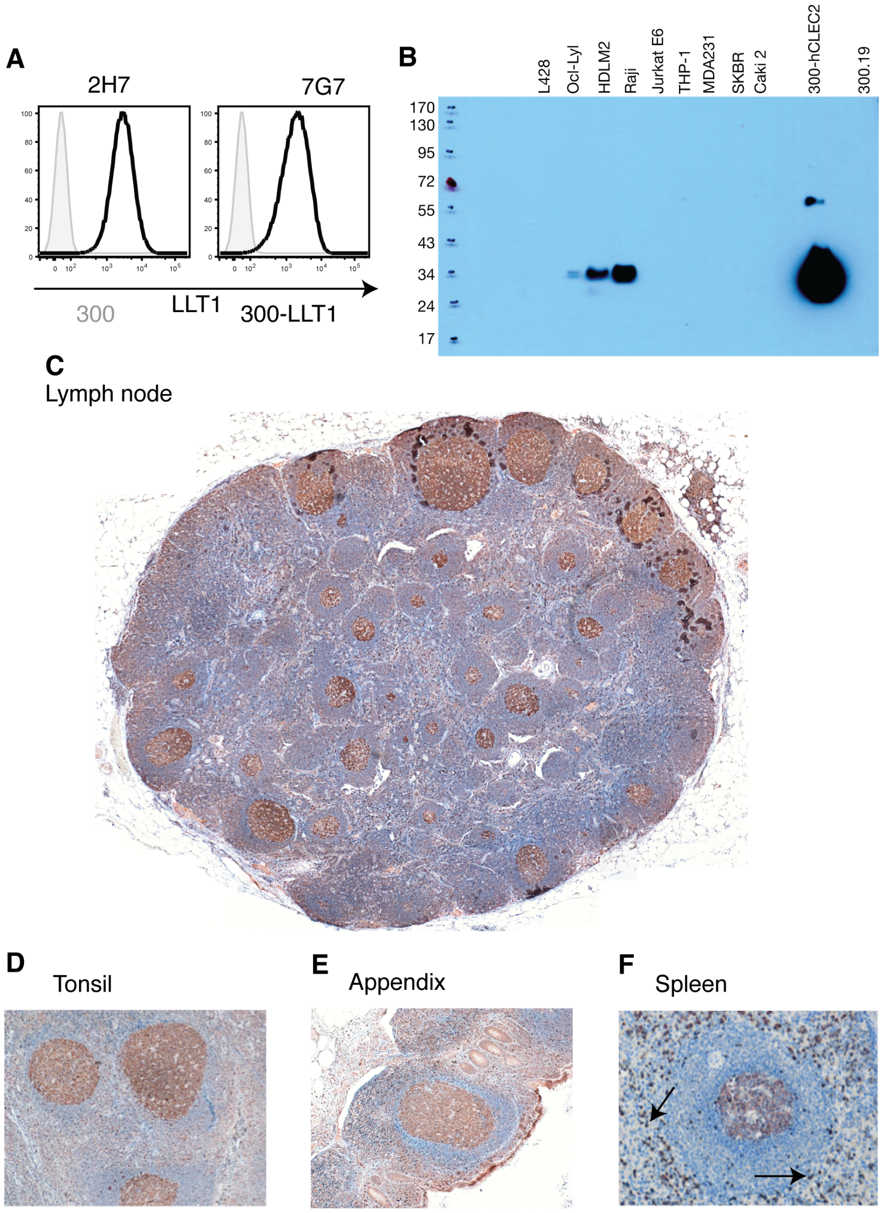

Figure S1. Germinal Centers of different secondary lymphoid organs are all positive for LLT1. (A) shows specific staining of the 2H7 and 7G7 clones using transfected human LLT1 300 cells. (B) Western blot assessment of 7G7 antibody specificity against a range of cell lines, the 2H7 clone was not functional by western blots (data not shown). (C-F) Tissue sections stained for LLT1 using the polyclonal goat anti-LLT1 (AF3480) antibody: (C) (x40) composite image from a lymph node, (D) tonsil (x20), (E) (x10) appendix, (F) spleen (x20), arrows point LLT1+ cells outside the germinal center.

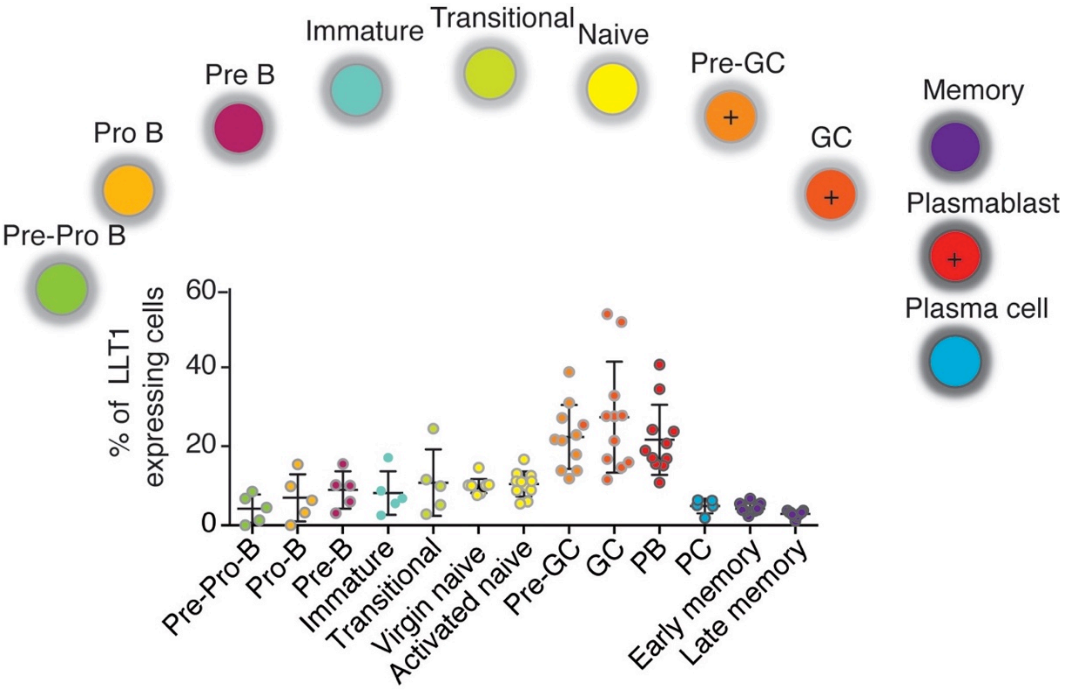

Figure S2. **LLT1 is expressed in the stages of pre-GC, GC B cells and plasmablasts.** LLT1 levels were analysed by FACS (2H7 clone) in different stages of B cell differentiation, using samples from bone marrow and tonsils. Pre-Pro B (CD10+ CD19- CD34+ IgM-), Pro-B (CD10+ CD19+ CD20- CD34+ IgM-), Pre-B (CD19+ CD20+ CD34-IgM-), Immature (CD10+ CD19+ CD20+ IgM+), Transitional (CD19+ CD20+ IgM++), Virgin naive (CD19+ IgD+ CD38-), Activated naive (CD19+ CD38+ IgD+), Pre-GC (CD19+, CD38++, IgD+), GC (CD19+ CD38 ++ IgD-), PB (CD19low, CD38+++, IgD-), PC (CD19low CD138+ IgD-), Early memory (CD19+ CD38+ IgD-) and late memory (CD19+ CD38- IgD-). LLT1 was not expressed on B cell precursors or transitional B cells, nor in tonsillar naive B cells. However, LLT1 was highly expressed in pre-GC and GC B cells from tonsils. Its levels remained high in plasmablasts but were quickly downregulated in memory B cells. Terminally differenatiated plasma cells did not express LLT1. (Bone marrow n=5; Tonsils n=10).

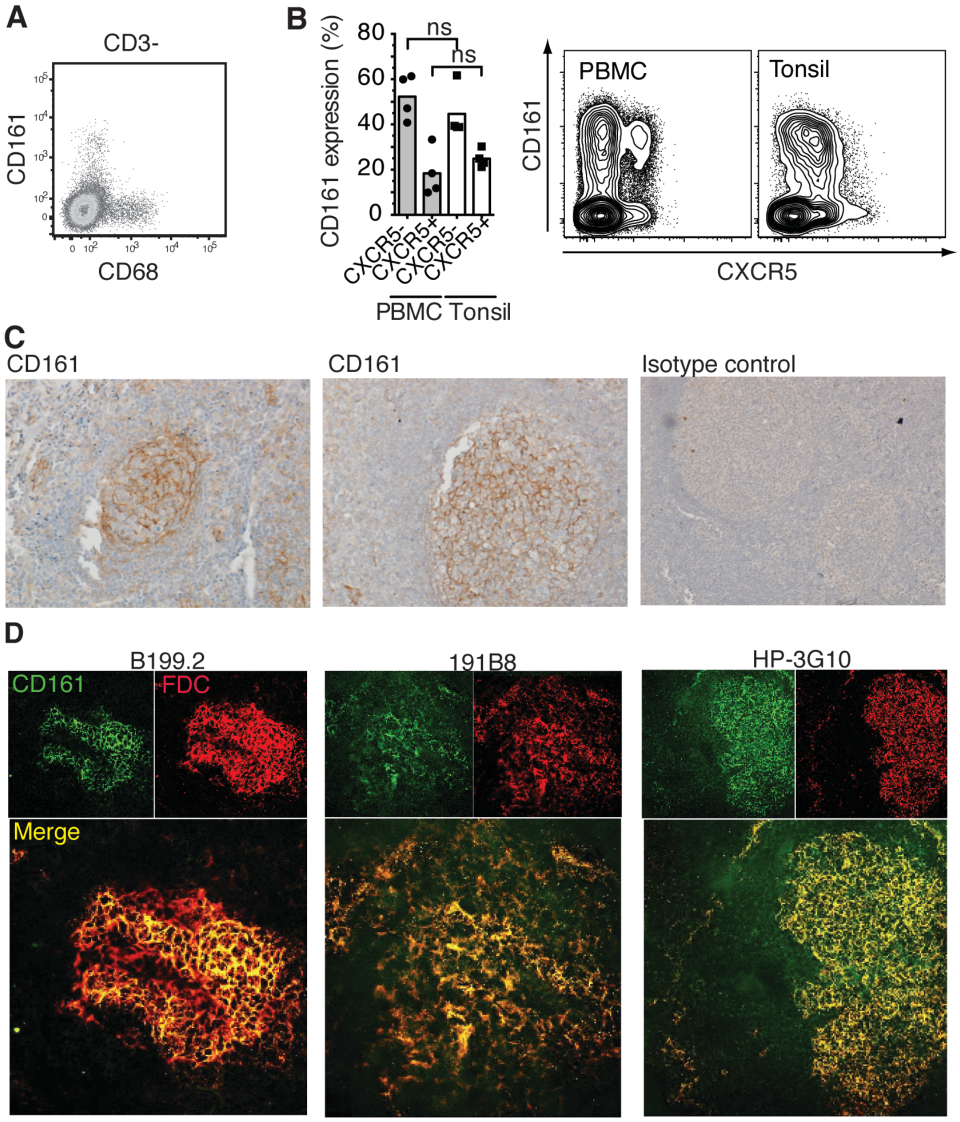

Figure S3. CD161 is expressed in the germinal center environment. (A) CD68 staining showed no expression CD161 macrophage (including the TBM population). (B) CD161 expression was lower on CXCR5+ CD4+T cells from both tonsils and PBMC. (C) Staining with anti-CD161 antibody (brown; B199.2 clone) of FFPE human tonsillar tissue (CD161 20x, Isotype control 10x) revealed an expression pattern similar to the one of FDCs. Representative images of one out of 3 independent experiments are shown. (D) Immunofluorescent staining of frozen human tonsils with the FDC marker and different anti-CD161 antibody clones (191B8, B199.2 and HP-3G10) revealed expression of CD161 on FDCs.

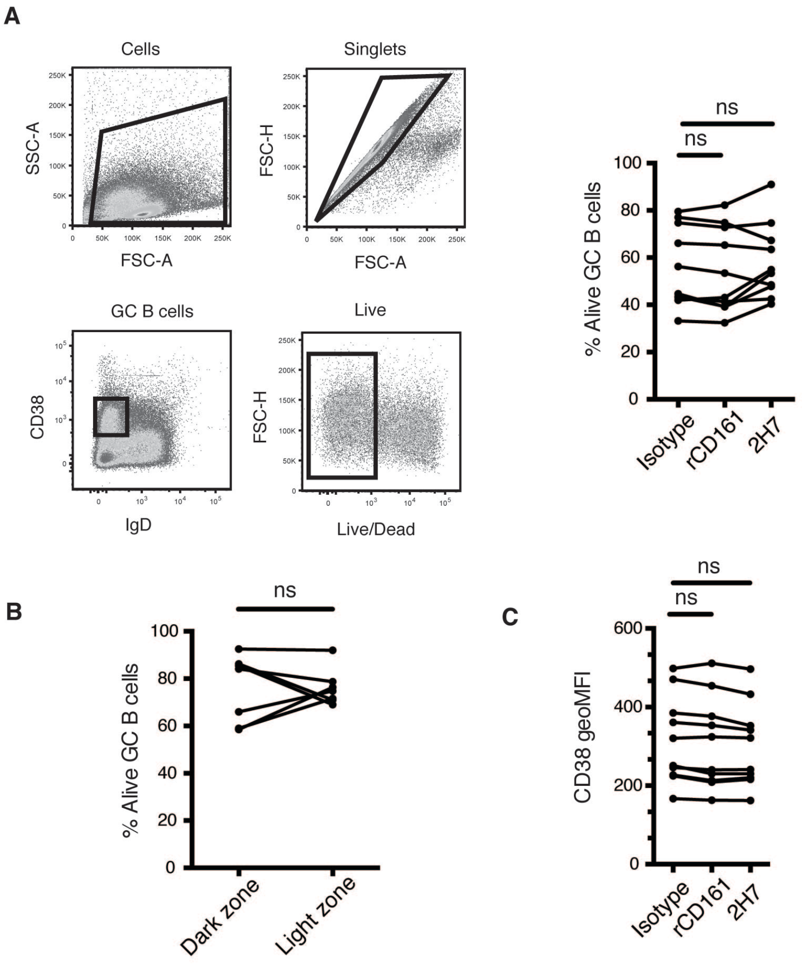

Figure S4. Controls for Figure 5. (A) The percentage of live GC B cells after overnight stimulation is shown. Purified B cells from tonsils were incubated with rCD161, 2H7 antibody or isotype control (n=10). No significant (ns) differences were detected between any of the groups (two-way ANOVA using a Bonferroni multiple comparisons test). (B) Sorted DZ and LZ B cells (Alive CD19+, CD83high/low CXCR4high/low) were rested overnight and the percentage of live B cells measured by flow cyometric analysis (n=7). No significant differences could be detected (non-parametric paired T-test). (C) Purified B cells from tonsils were incubated with rCD161, 2H7 antibody or isotype control, and levels of CD38 expression measured by flow cytometry (n=10). No significant differences were detected between any of the groups (two-way ANOVA using a Bonferroni multiple comparisons test).
